# Supplementary material for: Sequential Endoluminal Gemcitabine and Cabazitaxel with Intravenous Pembrolizumab as a Bladder-Preserving Strategy for Docetaxel-Unresponsive Non-Muscle Invasive Urothelial Carcinoma Following Transurethral Resection of Bladder Tumor
Source: Cancers (Basel). 2024 Jul 17;16(14):2561. doi: 10.3390/cancers16142561 (PMC11274621; doi:10.3390/cancers16142561)
Supplement: Supplementary file 1 [file cancers-16-02561-s001.zip › cancers-3095287-supplementary.pdf]

Supplementary Materials

Sequential Endoluminal Gemcitabine and Cabazitaxel with Intravenous Pembrolizumab as a Bladder-Preserving Strategy for Docetaxel-Unresponsive Non-Muscle Invasive Urothelial Carcinoma Following Transurethral Resection of Bladder Tumor

Ian M. McElree, Vignesh T. Packiam, Ryan L. Steinberg, Helen Y. Hougen, Sarah L. Mott, Mohamad Abou Chakra, Yousef Zakharia and Michael A. O'Donnell

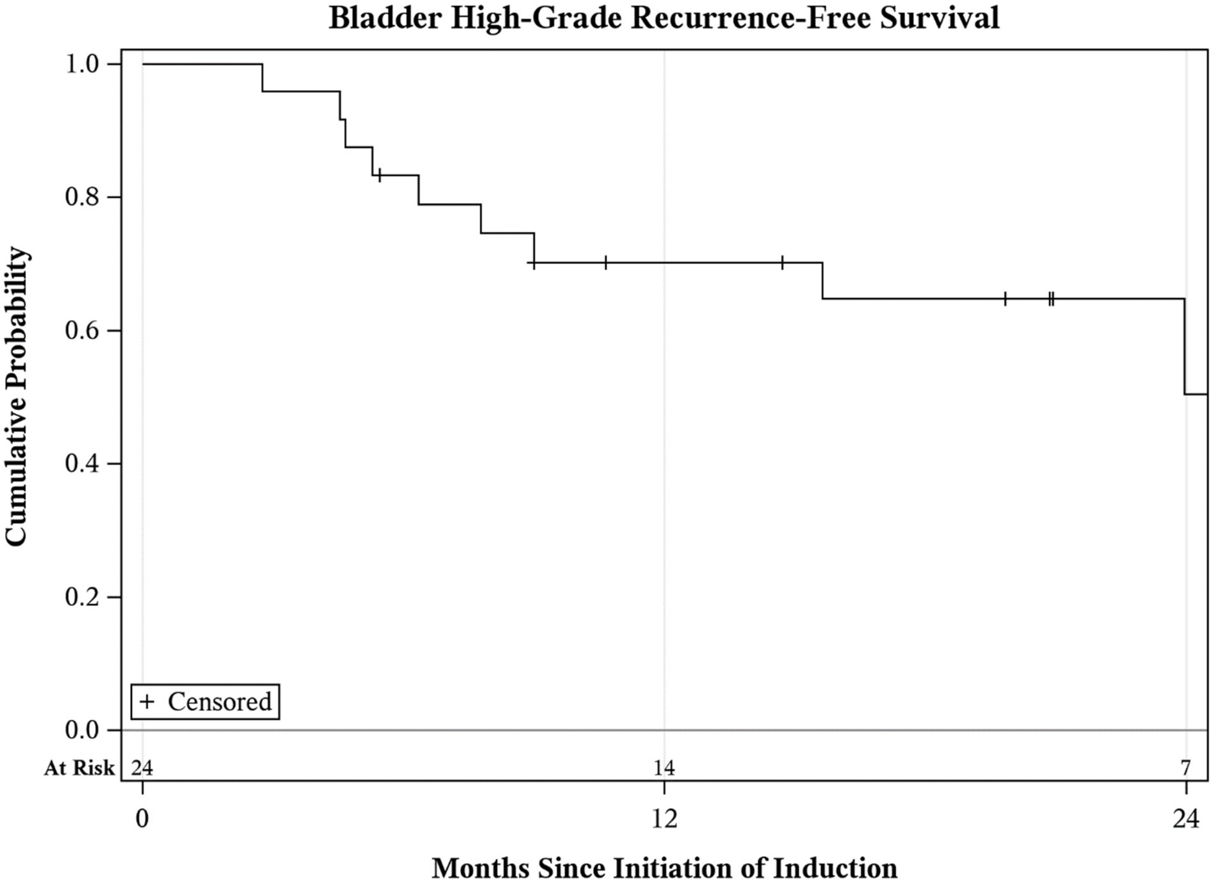

12 Months	24 Months
70% (47-85%)	50% (26-71%)

Figure S1. High-Grade Recurrence-Free Survival Among Patients Presenting with Bladder Cancer Following GCP Treatment.

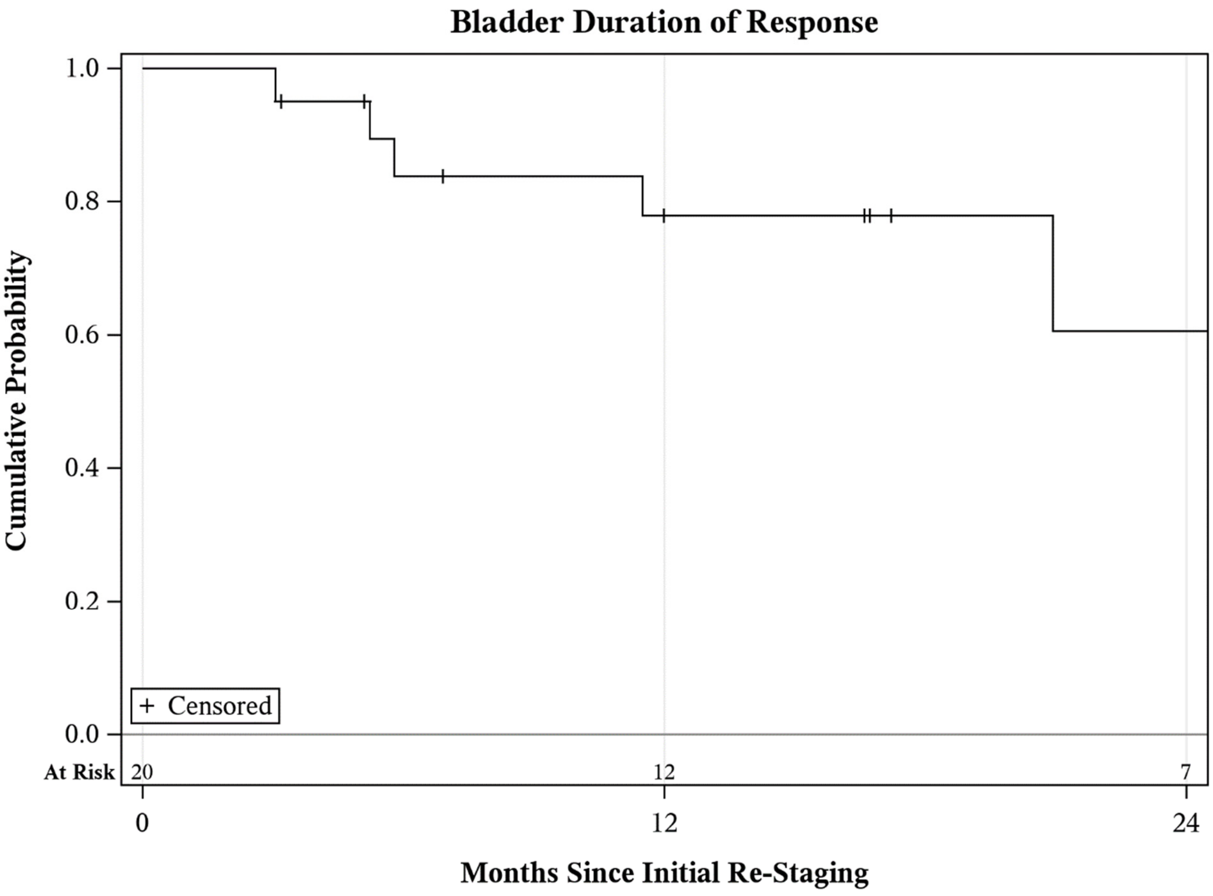

Figure S2. Duration of Response Following GCP Treatment.

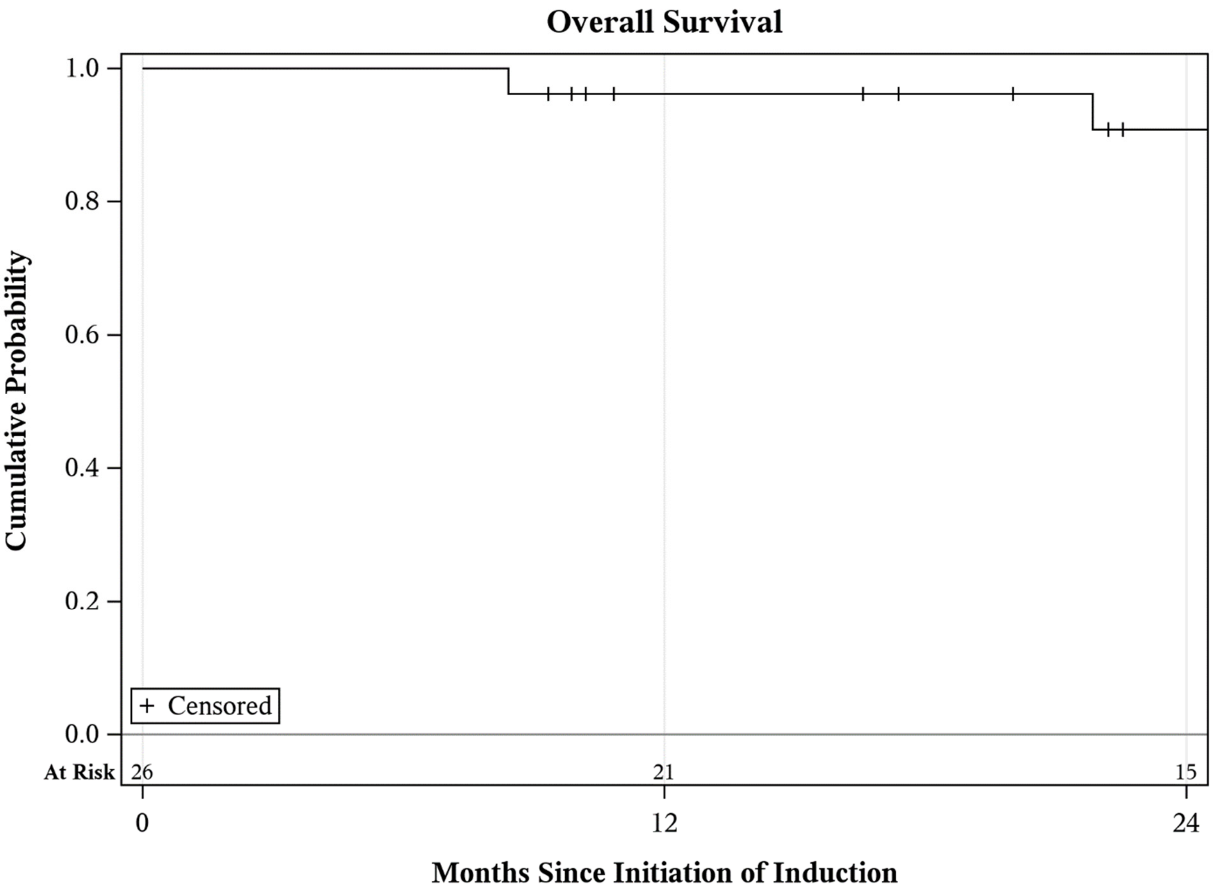

12 Months	24 Months
96% (76-99%)	91% (67-98%)

Figure S3. Overall Survival Following GCP Treatment.

Table S1. Clinical and pathological features of patients receiving cystectomy and Nephroureterectomy.

Specimen Type	Treatment Location*	Presenting Pathology	Prior Prostatic UC	Neoadjuvant Chemotherapy	Post-Surgical Pathology	PU disease at cystectomy
Cystectomy	Bladder	HG Cytology	No	No	pT2N1	Yes
Cystectomy	Bladder	CIS	Yes	Yes	pT2bN0	No
Cystectomy	Bladder	T1HG + CIS	Yes	No	pT0N0	No
Cystectomy	Bladder	HG Cytology	No	No	pTisN2	Yes
Cystectomy	Bladder	TaHG	No	No	pTisN0	No
Cystectomy	Bladder	CIS	No	No	pT2N0	Yes
Nephroureterectomy	Upper Tract	TaHG	No	No	pT1N0	-
Nephroureterectomy	Bladder	CIS	No	No	pT2N0	-

*Designates the location of original urothelial carcinoma treated with Gem/Cabaz therapy.

Table S2. Diagnostic and Recurrence Criteria for Additional Upper Tract Units Treated with GCP but Not Meeting Study Inclusion Criteria.

	Treatment Criteria			Recurrence Criteria			
**ID#**	**Prior Treatment**	**Cytological Results**	**FISH Results**	**Time to Recurrence**	**Cytological Results**	**FISH Results**	**Follow-up (months)***
1	Yes	Atypical	-	-	-	-	33
2	No	Suspicious	-	27 months	Negative	25/25 abnormal	28
3	No	Suspicious	-	-	-	-	32
4	Yes	-	39/40 abnormal	35 months	Positive HG	-	35
5	Yes	Suspicious	-	-	-	-	12
6	No	Atypical	25/25 abnormal	-	-	-	15
7	No	Atypical	30/50 abnormal	-	-	-	7

*Follow-up for recurrence.
